# Non-Myeloablative Chemotherapy as Consolidation Strategy After High-Dose Methotrexate-Based Chemoimmunotherapy in Patients With Primary CNS Lymphoma: A Retrospective Single Center Study in China

**DOI:** 10.3389/fonc.2022.792274

**Published:** 2022-02-23

**Authors:** Xuefei Sun, Yuchen Wu, Ruixian Xing, Xueyan Bai, Jun Qian, Hong Zhu, Qu Cui, Yuedan Chen, Qing Liu, Wenyuan Lai, Junhong Li, Yaming Wang, Shengjun Sun, Chunji Gao, Nan Ji, Yuanbo Liu

**Affiliations:** ^1^ Department of Hematology, Beijing Tiantan Hospital, Capital Medical University, Beijing, China; ^2^ Department of Neurosurgery, Xuanwu Hospital, Capital Medical University, Beijing, China; ^3^ Neuroimaging Center, Beijing Tiantan Hospital, Capital Medical University, Beijing, China; ^4^ Department of Hematology, Chinese PLA General Hospital, Beijing, China; ^5^ Department of Neurosurgery, Beijing Tiantan Hospital, Capital Medical University, Beijing, China

**Keywords:** PCNSL = primary central nervous system lymphoma, HD-MTX, consolidation, chemoimmunotherapy, R-MAD regimen

## Abstract

Primary central nervous system lymphoma (PCNSL) remains a disease with poor outcome and high recurrence rate. We retrospectively analyzed the clinical data of 243 immunocompetent patients with PCNSL in Beijing Tiantan Hospital. The median age of PCNSL patients was 57 years (range 10-95 years). For induction therapy, 94.7% of patients received high-dose methotrexate (HD-MTX) containing regimens, and 59.3% received rituximab, which increased over time. The overall response rate was 72.8%, with 58.8% achieving complete response. With a median follow-up of 27.0 months (95% confidence interval 23.6-30.4), the median progression-free survival (PFS) time was 14.0 months (95% CI 9.45-18.55), and the 2-year PFS rate was 33.2%. The median overall survival (OS) was not reached (NR), with an estimated overall survival rate at 4 years of 61.6%. Among 95 patients who completed sequential consolidation chemotherapy with either pemetrexed or etoposide plus cytarabine, the median PFS was 28 months (95% CI 17.11-38.89), and the estimated overall survival at 4 years was 78.7%. In conclusion, HD-MTX based induction chemotherapy with non-myeloablative sequential consolidation chemotherapy is an alternative feasible treatment option.

## Introduction

Primary central nervous system lymphoma (PCNSL) is an uncommon extranodal non-Hodgkin lymphoma, and more than 95% of PCNSLs are large B-cell subtypes. PCNSL in immunocompetent patients is distinct from systemic lymphoma in diagnosis, prognosis and therapeutic strategies (1). A population-based study reported a 5-year overall survival (OS) of 38-56% (2, 3).

High-dose methotrexate (HD-MTX) based systemic chemotherapy is widely accepted in current practice, meanwhile, promising results have been shown in several clinical trials investigating combination regimens of rituximab with cytotoxic chemotherapy (4–8). Although the importance of HD-MTX in newly developed PCNSL has become a general consensus, opinions on the optimal first-line chemotherapy regimens have not come to an agreement. While PCNSL responds to chemotherapy, about 50% of cases experience relapse in the first two years (9), emphasizing the necessity for efficient consolidation therapies. The use of whole-brain radiotherapy (WBRT) is limited by its neurotoxicity, especially in elderly patients. High-dose chemotherapy followed by autologous stem cell transplant (HCD-ASCT), however, is not available everywhere and is not suitable for elderly patients either. As a result, non-myeloablative sequential chemotherapy was considered another consolidation treatment strategy (5).

The object of this research was to summarize the characteristics among 243 PCNSL patients and analyze the efficacy of HD-MTX based induction therapy, meanwhile, explore the feasibility of nonmyeloablative sequential consolidation chemotherapy.

## Methods

### Patients

The clinical data of 243 immunocompetent patients with PCNSL from 10 May 2010 to 9 January 2020 were analyzed retrospectively. The inclusion criteria were as follows (1): PCNSL diagnosed by stereotactic biopsy, surgery or cerebrospinal fluid (CSF)/vitreous biopsy; (2) negative full-body CT scan or FDG-PET scan; and (3) immunocompetence and negative HIV status. Patients with primary intraocular lymphoma were excluded. The database was approved by the Beijing Tiantan Hospital Ethics Committee, and all patients gave written informed consent.

### Evaluation of Response

Response was assessed according to International PCNSL Collaborative Group criteria. Enhanced MRI was routinely evaluated before each induction or consolidation treatment or any sign of potential disease progression. The overall objective response rate was defined as the sum of the complete response (CR), unconfirmed complete response (CRu) and partial response (PR) rates. Progression-free survival (PFS) was calculated from the start of treatment to the time of disease progression or death due to PCNSL. Overall survival (OS) was calculated from the date of diagnosis to the time of death from any cause.

### Statistical Analysis

Clinical characteristics of patients with various induction regimens were compared using a chi square test. Probability estimates for PFS and OS were calculated by using Kaplan-Meier analysis. Cox’s regression model was applied for a multivariate survival analysis. SPSS statistics version 24.0 was utilized for all data analyses. P < 0.05 was considered significant.

### Data Sharing Statement

The datasets supporting the conclusions of this study are available from the corresponding author on reasonable request.

## Results

### Main Characteristics at Diagnosis

In total, 243 newly diagnosed PCNSL patients were included in the analysis. Their main characteristics at diagnosis are reported in **Table 1**. The median age was 57 years (range 10-95), and the median ECOG-PS (Eastern Cooperative Oncology Group performance status) score was 2 (range 0-4). Motor/sensory deficit and intracranial hypertension were predominant in 49.8% and 46.9% of the cases, respectively. Ocular involvement was diagnosed in 17 out of 119 patients who underwent ophthalmologic tests. Regarding diagnostic approaches, 94.7% received cerebral biopsy or tumor resection, and the remaining patients were diagnosed through vitrectomy, CSF cytology.

**Table 1:** Main characteristics at diagnosis.

	Median (range) or n/N (%)
No	243
Age, y	57 (10-95)
10-50	77 (31.7)
51-60	70 (28.8)
61-70	64 (26.3)
71-80	25 (10.3)
>80	7 (2.9)
Sex, M/F	138/105 (1.31)
ECOG-PS score	2 (0-4)
Elevated serum LDH	81/243 (33.3)
Symptoms at diagnosis	
Cognitive impairment	69 (28.4)
Walking disorders	37 (15.2)
Motor/sensory deficit	121 (49.8)
Intracranial hypertension	114 (46.9)
Epilepsy	8 (3.3)
Blurred vision	35 (14.4)
Ophthalmologic workup done	119 (49)
Ocular involvement	17
CSF workup done	76 (31.3)
CSF protein>0.5 g/L	45/76 (59.2)
CSF cell count>5 cell	32/76 (42.1)
Diagnostic method	
Cerebral biopsy	178 (73.3)
Tumor resection	52 (21.4)
Vitrectomy	4 (1.6)
CSF	9 (3.7)
Histopathologic diagnosis	243
Diffuse large B-cell lymphoma	196 (80.7)
Other high-grade B-cell lymphoma	3 (1.2)
Unclassifiable B-cell lymphoma	41 (16.9)
T-cell lymphoma	1 (0.4)
NK-T lymphoma	1 (0.4)
Intravascular large B-cell lymphoma	1 (0.4)
MRI	
Multiple lesions	134 (55.1)
Infratentorial involvement	67 (27.6)

CSF, cerebrospinal fluid.

### Induction Treatment

The main characteristics of the induction treatment are reported in **Table 2**. A total of 94.7%(n=230) of the patients received HD-MTX-containing chemotherapy, 221 received at least 3g/m^2^ per injection. A total of 59.3% (144) used rituximab, with an increase in use over time (from 35.4% in 2010-2016 to 64.6% in 2017-2020).

**Table 2:** Treatment characteristics.

	Values
Delay first symptoms to treatment, d	33 (0-1824)
Other chemotherapy	13/243 (5.3%)
HD-MTX-based chemotherapy	230/243 (94.7%)
Characteristics of HD-MTX treatment	
Dose per injection, g/m^2^	3.5 (1.5-3.5)
Total number of injections	6 (1-10)
3-3.5 g/m^2^	221
Induction chemotherapy protocols including HD-MTX	230
M	13/230 (5.7%)
MAD	62/230 (27.0%)
R-MAD	108/230 (47.0%)
MADD	11/230 (4.8%)
R-MADD	36/230 (15.7%)
Use of rituximab	144/243 (59.3%)
Consolidation regimen	95
Pemetrexed and cytarabine	35/95 (36.8%)
Etoposide and cytarabine	60/95 (63.2%)

HD-MTX, high-dose methotrexate, M, Methotrexate; R, Rituximab; MAD, Methotrexate +Ara-C +Dexamethasone; MADD, Methotrexate+ Ara-C+ Dexamethasone+ liposomal doxorubicin.

The HD-MTX containing regimen in our research mainly included the HD-MTX single regimen, HD-MTX, cytarabine (Ara-C), and dexamethasone (MAD) with or without rituximab (RTX), HD-MTX, Ara-C, liposomal doxorubicin and dexamethasone (MADD) with or without rituximab (drug dose see in **Supplementary Data 1**). Intravitreal methotrexate injection (0.04ml/400ug) were given after 2-4 cycles of intravenous HD-MTX containing chemotherapy when performance status was improved. based on suggestion of experienced ophthalmologist during the intermission of systemic chemotherapy.

### Outcome of First-Line Treatment

**Figure 1:**
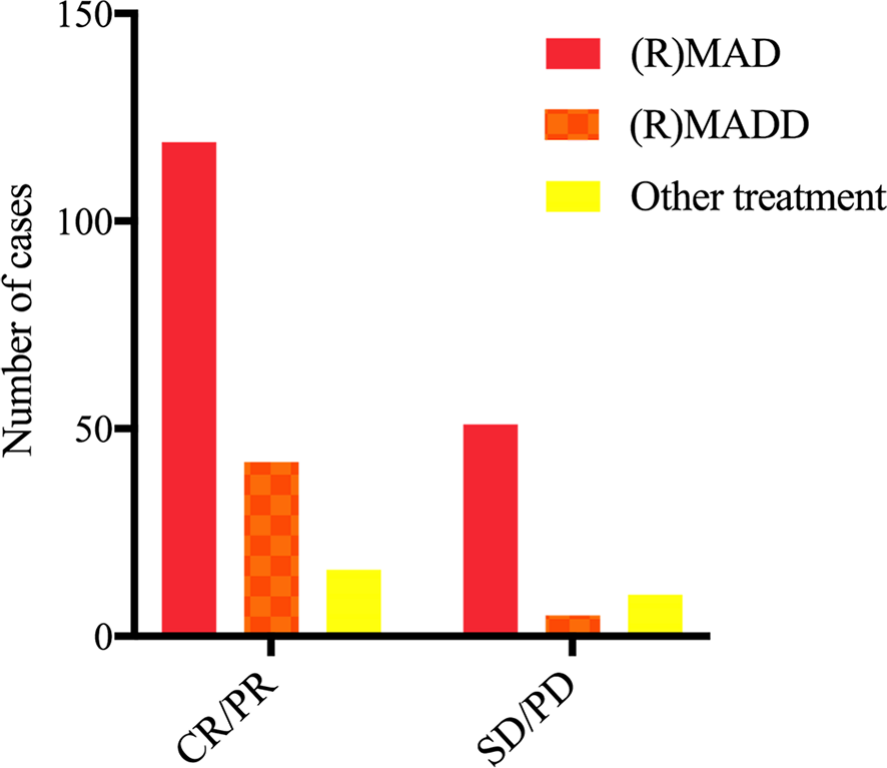
Response to first-line treatment.

**Figure 2:**
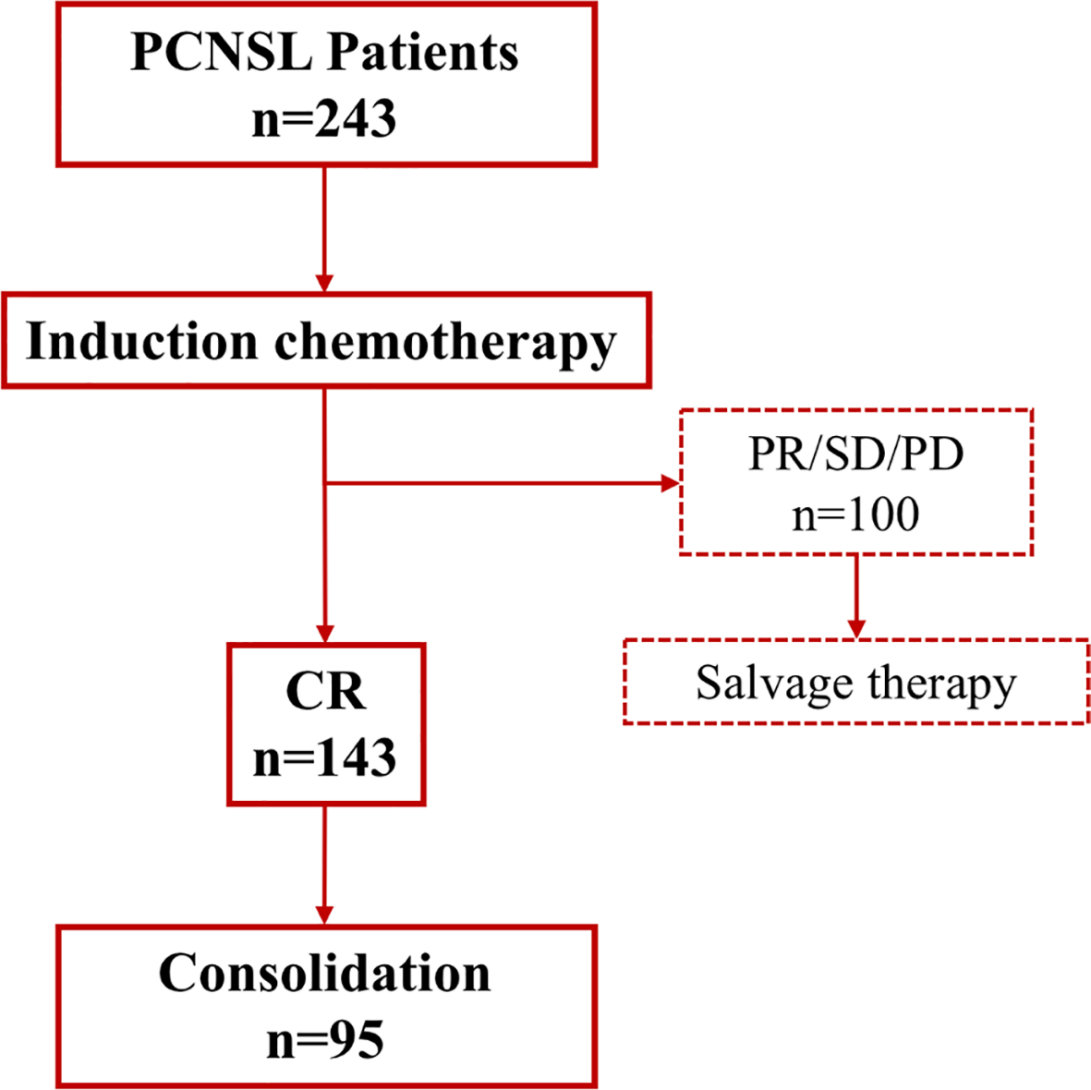
Response of induction and salvage treatment. CR, complete response; PR, partial response; PD, progressive disease; SD, stable disease.

The overall response rate (ORR) of all patients was 72.8%, with 58.8% achieving complete response after induction chemotherapy (IC). The proportion of disease progression was 20.2%. The combination of HD-MTX, cytarabine and dexamethasone-containing regimens with or without RTX was used in 70.0% of cases, and 19.3% added liposomal doxorubicin on the basis of the (R)MAD regimen (**Table 3**). While there was no significant difference in the use of RTX among (R)MAD and (R)MADD groups (p=0.498), the additional liposomal doxorubicin led to a higher ORR of 89.3% (**Figure 1**). Patients who didn’t achieve CR after induction therapy proceeded to salvage treatment (**Figure 2**).

**Table 3:** Outcome of first-line treatment.

Response	All patients (n=243)	Other treatment* (n=26)	(R)MAD (n=170)	(R)MADD (n=47)
Complete response	143 (58.8%)	10 (38.5%)	96 (56.5%)	37 (78.7%)
Partial response	34 (14.0%)	6 (23.1%)	23 (13.5%)	5 (10.6%)
Stable disease	17 (7.0%)	5 (19.2%)	11 (6.5%)	1 (2.1%)
Progression disease	49 (20.2%)	5 (19.2%)	40 (23.5%)	4 (8.5%)
Overall response rate	177 (72.8%)	16 (61.5%)	119 (70.0%)	42 (89.3%)

*Other treatment included HD-MTX single regimen, Temozolomide, Pemetrexed, lenalidomide regimen; (R)MAD, (Rituximab)+ Methotrexate +Ara-C +Dexamethasone; (R)MADD, (Rituximab)+ Methotrexate+ Ara-C+ Dexamethasone+ liposomal doxorubicin.

### Consolidation

Among 143 patients who achieved CR following induction chemotherapy, 95 patients proceeded to sequential consolidation chemotherapy. There was no statistical difference in age, gender and ECOG at diagnosis between patients received consolidation and those who didn’t (see in **Supplementary Data 2**). The lag time between induction therapy and consolidation was 2 months. Thirty-five patients received pemetrexed and the Ara-C regimen (PA), while sixty patients received etoposide and the Ara-C (EA) regimen. The PA regimen consisted of pemetrexed 900 mg/m^2^ administered on day 1, and then Ara-C was administered intravenously at 1-2 g/m^2^ on day 2 (oral folic acid was administered at 400μg daily 1 week before pemetrexed administration and continued for 3 weeks after the last dose). The EA regimen consisted of 100 mg/m^2^ etoposide on days 1-3 and 1-2 g/m^2^ Ara-C on days 2-3. The dose of Ara-C in both regimens depended on the patient age and ECOG-PS. Both sequential chemotherapy consolidation regimens were administered every 2 months for the first year and then every 6 months for the second year.

As expected, there’s no grade 4 toxicity in consolidation episode, 9.5% of patients experienced grade 2-3 neutropenia, 4.2% of patients experienced grade 2-3 thrombocytopenia, and 5.3% and 6.3% had grade 2 nephrotoxicity and hepatotoxicity, respectively. No grade 2-4 acute neurotoxicity was observed, detailed neurocognitive testing were not performed. There were no treatment-related deaths observed in our study (detailed toxicity see in **Supplementary Data 3**).

### Survival

The median follow-up period in 243 patients was 27.0 months (95% confidence interval 23.6-30.4), and the median PFS was 14.0 months (95% CI 9.45-18.55), with a 2-year PFS rate of 33.2%. The median OS was not reached (NR), with an estimated overall survival at 4 years of 61.6%. We compared the outcomes among (R)MAD, (R)MADD and other treatments. The median OS among all cases and all three groups was not reached, and the IC regimen was not significantly associated with OS (p=0.588). The median PFS was 18.00 (95% CI 10.73-25.27), 12.00 (95% CI 6.67-17.33), and 8.00 (95% CI 0.39-15.60) for (R)MADD, (R)MAD and other treatments, respectively, without statistical significance (p=0.177).

**Figure 3:**
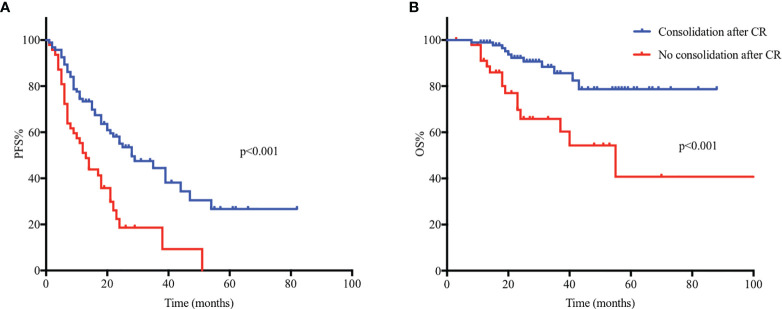
Kaplan-Meier analysis of progression-free survival (PFS) and overall survival (OS) in 143 PCNSL patients and comparison of PFS and OS between groups proceeded to consolidation (n=95) and who did not (n=48). **(A)** The median PFS was 28 months (95% CI 17.11-38.89) and 12 months (95% CI 7.74-16.26) (p < 0.001) for patients who completed sequential consolidation chemotherapy (n=95) and those who did not (n=48). **(B)** The median OS for patients who completed sequential consolidation chemotherapy (n=95) was not reached, for those who did not completed consolidation (n=48), the median OS was 55 months (95% CI 29.8-80.2).

For patients who completed sequential consolidation chemotherapy (n=95) and those who did not (n=48), the median PFS was 28 months (95% CI 17.11-38.89) and 12 months (95% CI 7.74-16.26) (p<0.001), respectively. The 2-year PFS for patients who received consolidation and those who did not was 54.5% and 18.2%, respectively. The median OS for patients who completed sequential consolidation chemotherapy (n=95) was not reached, for those who did not receive consolidation (n=48), the median OS was 55 months (95% CI 29.8-80.2). The estimated OS at 4 years was 78.7% and 54.3% (p<0.001), respectively (**Figure 3**).

### Prognostic Factors

**Figure 4:**
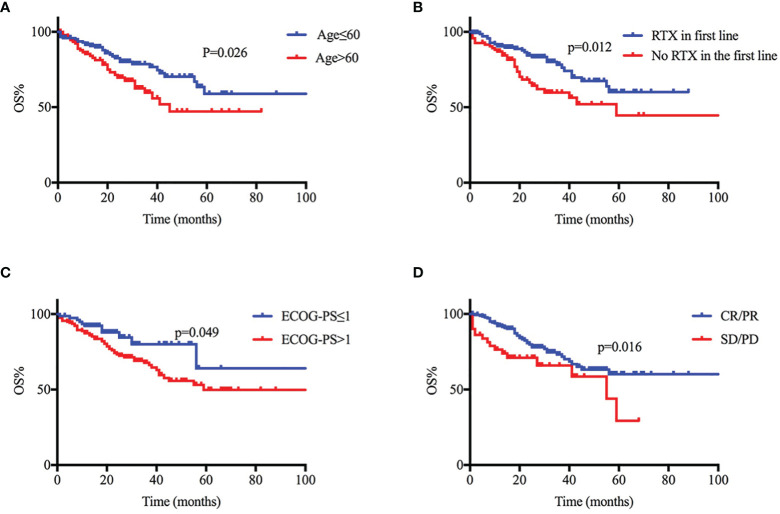
Clinical prognostic variables and their relationship to progression-free survival (PFS) and overall survival (OS). **(A-D)** Age ≤ 60, ECOG-PS ≤ 1, early response to induction chemotherapy (IC), and use of RTX were associated with prolonged OS in univariate analysis.

We included factors previously reported in other studies that have prognostic effects in PCNSL. Age, sex, ECOG-PS score, involvement of deep regions and serum LDH at diagnosis were factors in the IELSG score, but we did not include CSF protein because only 75/243 underwent CSF testing. The other main prognostic factors are indicated in **Table 4**. A better early response was found to be associated with a longer PFS time; however, we did not find a significant correlation between PFS and other clinical variables (see in **Supplementary Data 4**). Age ≤ 60, ECOG-PS ≤ 1, early response to IC, and use of RTX were associated with prolonged OS in univariate analysis, and all the above-mentioned factors, except for the ECOG-PS score, were associated with prolonged OS in multivariate analysis (**Figure 4**).

**Table 4:** Univariate and multivariate analyses of OS.

	N	Univariate analysis		Multivariate analysis	
		Median OS	P value	HR (95%CI)	P value
Age ≤ 60 y	147	NR	0.026	1.745 (1.024-2.973)	0.041
Age >60 y	96	45			
Male	138	NR	0.918		
Female	105	59			
ECOG-PS ≤ 1	79	NR	0.049	1.855 (0.940-3.664)	0.075
ECOG-PS>1	164	59			
Normal blood LDH	163	NR	0.608		
Elevated blood LDH	80	59			
Unifocal lesion	99	NR	0.359		
Diffuse lesion	144	NR			
Absence of deep structure involvement	76	56	0.293		
Deep structure involvement	167	NR			
Tumor resection	52	NR	0.508		
No tumor resection	191	NR			
RTX in first-line treatment	144	NR	0.012	2.183 (1.305-3.645)	0.003
No RTX in first-line treatment	99	59			
Doxorubicin in first-line treatment	47	NR	0.653		
No doxorubicin in first-line treatment	196	NR			
Early response			0.016	2.081 (1.168-3.706)	0.013
CR/PR	186	NR			
SD/PD	57	56			

PFS, progressive free survival; RTX, rituximab; CR, complete response; PR, partial response; SD, stable disease; PD, progressive disease; NR, not reached.

## Discussion

In this single-center retrospective study, we reported the clinical characteristics, outcomes and prognostic factors of 243 PCNSL patients. Our team previously reported on outcomes of the (R)MAD regimen, showing a CR rate of 66.7% and a 2-year PFS rate of 34.0%. In this study, despite the inclusion of patients with advanced age and poor performance status, a CR rate of 58.8% and a 2-year PFS rate of 33.2% were achieved, in line with our previous report (10). This hospital-based study could add to current clinical trials and better reflect real-world PCNSL outcomes and survival.

There is little uniformity in the management of PCNSL outside of the consensus regarding the use of HD-MTX is the absolute backbone of PCNSL induction therapy. Drugs combining HD-MTX are often dictated by geographical region and drug availability. The currently used polychemotherapy regimens have an ORR of 35-74% and confer a median OS of 25-50 months (5, 9, 11–13). A previous study by AJ Ferreri proved that the addition of HD-cytarabine to HD-MTX was associated with improved activity against PCNSL. This randomized phase 2 study included 79 newly diagnosed PCNSL patients, and the ORR of the MA group was 69%, with a median PFS of 18 months (9). Our team introduced a modified dosage on the basis of the MA regimen in a retrospective study with 60 PCNSL cases and achieved a CR rate of 53.3% and a 2-year PFS rate of 34.0% (10). Other prospective trials including MA with or without thiotepa or temozolomide led to an ORR range of 53.0-92.0% due to varying consolidation strategies (4, 5, 12). With the limited activity of the MA regimen and unavailability of thiotepa, we introduced liposomal doxorubicin into the induction chemotherapy regimen.

Although doxorubicin is pivotal for the treatment of systemic aggressive lymphoma, was absent from the treatment of PCNSL due to its poor penetration of the brain-blood barrier (14, 15). Therefore, the formulation of liposomal doxorubicin was introduced to overcome this barrier (14, 16–19). Lionakis et al (20) reported the efficacy of DA-TEDDi-R in PCNSL. They measured the plasma and CSF pharmacokinetics of liposomal doxorubicin after the first cycle of induction chemotherapy and found that the CSF concentration of liposomal doxorubicin was low but with measurable concentrations during the entire treatment cycle, which suggested an accumulation effect. According to another study based on human and murine intracerebral breast cancer, liposomal doxorubicin achieved 7–17-fold higher concentrations in tumors than in the normal brain (14, 17). In this analysis, the addition of liposomal doxorubicin led to a higher ORR and tended to achieve better efficacy and survival in PCNSL. A larger sample size will be needed to prove its potential efficacy in PCNSL.

The optimal consolidation for PCNSL has yet to be elucidated. There are multiple lines of evidence supporting whole-brain radiotherapy (WBRT) and myeloablative high-dose chemotherapy followed by autologous stem cell transplant (HDC-ASCT) (21–24). Delayed neurotoxicity associated with WBRT can be a risk especially for elderly patients, reduced-dose WBRT and ASCT can initially improve cognition but developed delayed neurotoxicity after 3 years (25). As a result, for a subset of patients, predominantly elderly patients who were not candidates for HCD-ASCT and were also more likely to be susceptible to neurocognitive toxicity by WBRT, nonmyeloablative sequential consolidation chemotherapy was a feasible option. Rubenstein et al (5) conducted a study of high-dose chemotherapy consolidation in PCNSL with etoposide plus cytarabine (EA) (CALGB 50202) offering another consolidation treatment option in PCNSL without episodes of grade 3 or 4 neurotoxicity in consolidation. In agreement with CALGB 50202 and a few other retrospective studies (26, 27), our results showed that nonmyeloablative consolidation chemotherapy was feasible in PCNSL patients who achieved CR after first-line IC. The 2-year time to progression was longer in the report of Rubenstein than ours, which may be due to the exclusion of patients with PS larger than 2 in clinical trials. In our research, similar to CALGB 50202 study, no sever acute neurotoxicity was observed, detailed neurocognitive test should be explored in further trails. There is an ongoing study comparing the efficacy of myeloablative versus non-myeloablative consolidation chemotherapy in PCNSL (NCT01511562 and NCT02531841), which may provide us with more evidence in the future.

Regarding prognostic factors, two prognostic scoring systems are widely used to predict the clinical outcome of PCNSL: International Extranodal Lymphoma Study Group (IELSG) scoring (28) and Memorial Sloan Kettering Cancer Center (MSKCC) scoring (29). Age and ECOG-PS score were well-established prognostic factors in both the scoring systems above, consistent with our results. However, the use of RTX has been highly debated; in our study, administration of RTX was a favorable prognostic factor. The HOVON 105/ALLG NHL 24 trial did not observe a beneficial effect of RTX, while in IELSG32, another randomized trial, the HR for progression-free survival was 0.52 (95% CI 0.32–0.86) and for overall survival, it was 0.63 (0.42–1.02), both strongly supporting rituximab. Despite the controversy of the benefit of RTX, in most first-line studies, rituximab has remained an indispensable component of treatment for patients with primary CNS lymphoma (9, 30, 31). Serum LDH, deep structure involvement, and tumor resection were not related to outcome. With the evolution of PCNSL treatment, drugs such as ibrutinib have been included in the treatment of relapsed/refractory and even newly diagnosed PCNSL; thus, in the new drug era, new prognostic scoring systems should be explored.

This study has few limitations. First, this is a retrospective study, and 23 patients were lost to follow-up, but this number did not exceed 10% of our sample size. Induction treatment option may cause bias for patients with stronger treatments tended to have better financial situation and better tolerance to treatment. In the analysis of prognostic factors, immunohistological information on BCL-2, MYC, BCL-6 and MMSE was not included (32, 33). Data on detailed and delayed neurotoxicity and quality of life need further evaluation. Validation for the efficacy of EA sequential consolidation chemotherapy in prospective studies is needed in the future.

In conclusion, high-dose methotrexate (HD-MTX)-based induction chemotherapy with non-myeloablative sequential consolidation chemotherapy was a feasible strategy for PCNSL patients. Age and early treatment response were independent predictors of survival. Further prospective clinical trials should be conducted.

## Data Availability Statement

The raw data supporting the conclusions of this article will be made available by the authors, without undue reservation.

## Ethics Statement

Ethical approval was provided by Beijing Tiantan Hospital Ethics Committee, Capital Medical University (Ethical approval reference number: KY 2020-066-02). Informed consent was written obtained when patients were admitted to Department of Neurosurgery or Department of Hematology before initiation of chemotherapy. Written informed consent to participate in this study was provided by the participants’ legal guardian/next of kin.

## Author Contributions

YL designed the study. NJ, YMW, and CG provided the patient samples. SS revised neuroimaging. XS and YCW analyzed the data and wrote the manuscript. RX, QC, HZ, and JQ performed the experiments. XB, YC, QL, JL, and WL collected and analyzed the data. All the authors have read the manuscript and approved its submission.

## Funding

This study was supported by the Capital’s Funds for Health improvement and Research (NO. 2020-2-2049).

## Conflict of Interest

The authors declare that the research was conducted in the absence of any commercial or financial relationships that could be construed as a potential conflict of interest.

## Publisher’s Note

All claims expressed in this article are solely those of the authors and do not necessarily represent those of their affiliated organizations, or those of the publisher, the editors and the reviewers. Any product that may be evaluated in this article, or claim that may be made by its manufacturer, is not guaranteed or endorsed by the publisher.
