# Supplementary material for: Non-Myeloablative Chemotherapy as Consolidation Strategy After High-Dose Methotrexate-Based Chemoimmunotherapy in Patients With Primary CNS Lymphoma: A Retrospective Single Center Study in China
Source: Front Oncol. 2022 Feb 23;12:792274. doi: 10.3389/fonc.2022.792274 (PMC8904393; doi:10.3389/fonc.2022.792274)
Supplement: Supplementary file 1 [file DataSheet_1.pdf]

Supplementary material 1

Induction Chemotherapy

1. (R)MAD regimen:

Day 0: Rituximab was administered at 375 mg/m^2.

Day 1: Methotrexate 3.5 g/m^2 IV within 3.5 hours.

Day 2: Ara-C IV (1–2) g/m^2; Leucovorin rescue every 6 hours until the methotrexate < 0.10 μmol/L.

Day 1-3: Dexamethasone (5-10)mg IV.

2. (R)MADD regimen:

Day 0: Rituximab was administered at 375 mg/m^2.

Day 1: Methotrexate 3.5 g/m^2 IV within 3.5 hours.

Day 2: Ara-C IV (1–2) g/m^2; Leucovorin rescue every 6 hours until the methotrexate < 0.10 μmol/L.

Day 3: liposomal doxorubicin IV 20–25 mg/m^2.

Day 1-3: Dexamethasone (5-10)mg IV.

The dose of Ara-C and HD-MTX depended on the patient age and Eastern Cooperative Oncology Group (ECOG) performance status. The induction treatment consisted of 6 cycles of chemotherapy at 3-week intervals between cycles.

Consolidation chemotherapy

1. EA regimen

Day 1-3: Etoposide IV 100mg/m^2.

Day 2-3: Ara-C IV (1–2) g/m^2.

2. PA regimen

Day 1: Pemetrexed IV $900mg/m^2$, oral folic acid 400ug daily 1 week before pemetrexed and continued for 3 weeks after last dose.

Day 2-3: Ara-C IV (1–2) g/m^2.

The dose of Ara-C depended on the patient age and Eastern Cooperative Oncology Group (ECOG) performance status. Both sequential chemotherapy consolidation regimens were administered every 2 months for the first year and then every 6 months for the second year.

Supplementary material 2

Characteristics	Consolidation after CR(n=95)	Non-consolidation after CR(n=48)	P value
Age≤60 y	63	34	0.705
Age >60 y	32	14	
Male	60	28	0.590
Female	35	20	
ECOG≤1	37	14	0.273
ECOG>1	58	34	

Supplementary material 3

Grade 2-3 toxicity in consolidation

	Grade 2		Grade 3		N=95
	EA	PA	EA	PA	
Neutropenia	5	1	3	0	9(9.5%)
Thrombocytopenia	3	0	1	0	4(4.2%)
Anemia	0	0	0	0	0
Febrile neutropenia or infections	0	0	0	0	0
Nephrotoxicity	4	1	0	0	5(5.3%)
Hepatotoxicity	5	1	0	0	6(6.3%)
Cardiotoxicity	0	0	0	0	0
Acute neurotoxicity	0	0	0	0	0

EA, Etoposide+ cytarabine; PA, Pemetrexed+ cytarabine

Supplementary material 4

Univariate and multivariate analyses of PFS

	Univariate analysis		
	N	Median PFS	P value
Age ≤60 y	147	14.0	0.382
Age >60	96	10.0	
Male	138	11.0	0.547
Female	105	16.0	
ECOG≤1	79	16.0	0.607
ECOG>1	164	12.0	
Normal blood LDH	163	15.0	0.216
Elevated blood LDH	80	9.0	
Unifocal lesion	99	21.0	0.055
Diffuse lesion	144	10.0	
Absence of deep structure involvement	76	13.0	0.390
Deep structure involvement	167	14.0	
No tumor resection	52	16.0	0.383
Tumor resection	191	11.0	
Doxorubicin in first line	47	18.0	0.127
No doxorubicin in first line	196	11.0	
RTX in first line	144	16.0	0.070
No RTX in the first line	99	11.0	
Early response			<0.001
CR/PR	186	18.0	
SD/PD	57	5.0	

PFS, progressive free survival; RTX, rituximab; CR, complete response; PR, partial response; SD, stable disease; PD, progressive disease.
